# Intracluster correlation coefficients for the Brazilian Multicenter Study on Preterm Birth (EMIP): methodological and practical implications

**DOI:** 10.1186/1471-2288-14-54

**Published:** 2014-04-22

**Authors:** Giuliane J Lajos, Samira M Haddad, Ricardo P Tedesco, Renato Passini Jr, Tabata Z Dias, Marcelo L Nomura, Patrícia M Rheder, Maria H Sousa, Jose G Cecatti

**Affiliations:** 1 Department of Obstetrics and Gynecology, School of Medical Sciences, University of Campinas, Campinas, Brazil; 2 Center for Studies in Reproductive Health of Campinas (Cemicamp), Campinas, Brazil

**Keywords:** Intracluster correlation coefficient, Preterm birth, Spontaneous preterm labor, Premature rupture of membranes, Indicated preterm delivery, Neonatal morbidity

## Abstract

**Background:**

Cluster-based studies in health research are increasing. An important characteristic of such studies is the presence of intracluster correlation, typically quantified by the intracluster correlation coefficient (ICC), that indicate the proportion of data variability that is explained by the way of clustering. The purpose of this manuscript was to evaluate ICC of variables studied in the Brazilian Multicenter Study on Preterm Birth.

**Methods:**

This was a multicenter cross-sectional study on preterm births involving 20 referral hospitals in different regions of Brazil plus a nested case–control study to assess associated factors with spontaneous preterm births. Estimated prevalence rates or means, ICC with 95% confidence intervals, design effects and mean cluster sizes were presented for more than 250 maternal and newborn variables.

**Results:**

Overall, 5296 cases were included in the study (4,150 preterm births and 1,146 term births). ICC ranged from <0.001 to 0.965, with a median of 0.028. For descriptive characteristics (socio-demographic, obstetric history and perinatal outcomes) the median ICC was 0.014, for newborn outcomes the median ICC was 0.041 and for process variables (clinical management and delivery), it was 0.102. ICC was <0.1 in 78.4% of the variables and <0.3 for approximately 95% of them. Most of ICC >0.3 was found in some clinical management aspects well defined in literature such as use of corticosteroids, indicating there was homogeneity in clusters for these variables.

**Conclusions:**

Clusters selected for Brazilian Multicenter Study on Preterm Birth had mainly heterogeneous findings and these results can help researchers estimate the required sample size for future studies on maternal and perinatal health.

## Background

Cluster-based studies involving aggregated units such as hospitals, health centers, schools or medical practices are increasingly being used in healthcare evaluation, especially in cluster randomized trials, which are perhaps the most high impact form of public health research/evaluation study design that can benefit from good extent estimates of ICC. In such situations, population groups (specific geographical areas), healthcare units (hospitals) or healthcare sectors are considered primary sampling units and generally all subjects belonging to each group are included to obtain data of interest [1,2].

However, depending on the method of selection, data obtained from clusters may not be sufficiently representative to allow for generalization. Population observed in clusters can present a large degree of similarity in some characteristics (homogeneity), unlike when there is a simple random sampling (SRS), in which each individual has the same probability of being selected in the general population, with more heterogeneity [2].

Therefore, an important characteristic of cluster-based studies is to evaluate the proportion of data variability that is explained by means of clustering, and this reliability may be analyzed by measuring inter and intracluster variance [3].

Intracluster correlation coefficient (ICC), denoted by ρ, is defined as the ratio of the between-cluster variance to the total variance (both between and within clusters), and therefore has a value between 0 and 1 [4,5]. Its value depends on the type of variable, cluster size and the prevalence of the condition [6]. Coefficients close to zero indicate that individuals within clusters are no more similar to each other than individuals from different clusters (the variable is randomly distributed among clusters); otherwise the values close to 1 reflect the homogeneity in a sample [7]. In other words, for cluster based population studies this heterogeneity (ICC close to zero) is desired as a proxy to the subjects being randomly selected.

The increase in variance due to clustering, compared to what would be obtained if sampling had been carried out by the SRS method, is calculated by design effect (Deff) [8]. It is given by 1 + (*m*-1) *ICC*, where *m* is the average cluster size [9]. Deff value is directly proportional to ICC and to the size of a cluster [10].

The ICC estimate in cluster studies is very useful for the development of new studies in the same field, because values obtained could be used as a correction factor for the calculation of sample size needed, thus avoiding underestimates, since in studies in which SRS is used, the sample size required to achieve sufficient statistical power is usually smaller [4].

The purpose of this manuscript is to evaluate the ICC of variables studied in the Brazilian Multicenter Study on Preterm Birth, a multicenter cross-sectional study on preterm births involving 20 referral hospitals in different regions of Brazil plus a nested case–control study. Estimated prevalence rates or means, ICC with 95% confidence intervals, design effects and average cluster sizes were also objectives for this study and they are presented for more than 250 maternal and neonatal variables.

## Methods

The Brazilian Multicenter Study on Preterm Birth consisted of a multicenter cross-sectional study plus a nested case–control study to assess their associated factors implemented in referral obstetrical units (clusters) from several states of the country. The full research proposal has already been published elsewhere [11].

A single-stage cluster sampling was used. Clusters were selected by an invitation to 27 healthcare institutions that build a national network called Brazilian Network for Studies on Reproductive and Perinatal Health. They are located in the five geographical regions of the country, almost all of them are public institutions, and all of them receive both low and high risk pregnant women. Initially 26 centers accepted to participate, but 20 selected institutions were able to fully take part in the study.

The sample size was calculated using the official prevalence of preterm births in Brazil of around 6.5% [12]. Considering an acceptable absolute difference of about 0.25% between the sample and the population prevalence, and a type I error of 5%, initial surveillance of a sample size of 37,000 deliveries was necessary. For the case–control study component, the estimated sample size was 1,055 women in each group (cases and controls). The total number of preterm births estimated to be followed in both components of the study was around 3,600.

The participating centers performed a prospective surveillance of all patients admitted to give birth in order to identify preterm births. For this purpose and according to standard international definitions, preterm birth was considered that occurring before 37 completed weeks of gestational age evaluated by an ultrasound scan performed early in pregnancy, by a known date of the last menstrual period, or alternatively by the evaluation of the somatic age of the newborn. During the first months of the study, in order to complete the sample for the appropriate analysis of the factors associated with spontaneous preterm birth, a random sample of women who had full-term birth was also selected.

Data was collected during six to twelve months for each center, from April 2011 to March 2012, in a detailed form called “Questionnaire” including 306 variables from four sources: interview with women in the postpartum period, medical records and prenatal chart of the mother (before hospital discharge), and newborn medical records (within sixty days after birth, even if it remained in hospital for longer period). An electronic system of data entry called *OpenClinica*® was selected and a proper clinical research form (CRF) was designed for the input of data after the questionnaire of each case was completed and reviewed.

High quality data and reliable information was guaranteed by several steps: preparatory meetings, development of detailed manuals of operation, monitoring technical site visits to the centers, close monitoring of data collection and data entry, concurrent query management, checking for logical inconsistencies, and correction of database. The research proposal was firstly approved by the Institutional Review Board of the coordinating center and then confirmed by IRB of each other participating center.

### Data analysis

In this study, each of the 20 participating centers (hospital) was considered a primary sampling unit (PSU) and there was no stratification of the PSU or weighting of the data. The subject (unit of analysis) was woman who delivered preterm (case) or at term (control).

Estimated prevalence (categorical variables) or means (continuous numeric variables), intracluster correlation coefficients (ICC), their respective 95% confidence intervals (CI), design effects (Deff) and mean cluster size of each variable were calculated. Software programs used for analysis were SPSS® version 20.0 [13] and Stata version 7.0 [14], taking into consideration the cluster sampling plan (centers) for data analysis.

According to Kish [2], ICC (*Roh*) is: ρ = (*s*^2^_*a*_ − *s*^2^_*b*_/*b*)*/s*ˆ^2^, where *s*^2^_*a*_ is the variance between clusters; *s*^2^_*b*_ is the variance within clusters, b is the size of clusters and *s*ˆ^2^ is the estimate of *S*^2^ (variance in individual level). The estimate *s*ˆ^2^ is obtained by: *s*ˆ^2^ = *s*^2^_*a*_ + [(*b* − 1)/*b*]*s*^2^_*b*_. Stata’s equivalent computing formula for ICC [14] is: ICC = [(*F* − 1)*a*/*n*]/1 + (*F* − 1)*a*/*n*, where ‘F’ is the Snedecor’s F-value from the ANOVA table and ‘a’ is the number of groups. The variance estimate for ICC is obtained by an extensive asymptotic formula and because this it was not showed.

For this study, the Design effect - DEFF [2] is *Deff* = var_actual_(*r*)/var_SRS_(*r*) = *s*^2^*a*/*a*/*s*^2^/*n*) where var_actual_(r) is the estimated variance according to the complex design being studied and var_SRS_(r) is the variance in the estimator considering the design as if it were calculated using a SRS of the same size, *n*.

## Results

During fifteen months, 5,296 births were included in the study, 4,150 of them being preterm births (1,491 due to spontaneous preterm labor, 1,191 due to a prelabor premature rupture of membranes and 1,468 due to a therapeutic interruption of pregnancy either for a maternal or fetal condition) and a sample of 1,146 term births to be used as controls for the case–control component.

Clustering was not stratified by region. Proportionally more centers were located in the Southeast of the country and consequently over half of births were from this region (11/20 – 53.5%). The other centers were from Northeast region (7/20 – 35%), contributing with 34.8% of births studied, South region (2/20 – 10%), with 11.7% of births. The mean size of each cluster was 265 cases.

### Estimated ICCs

Estimated ICCs are presented in Tables 1, 2, 3, 4, 5, 6, 7, 8, 9 and 10 for each of 261 variables. Tables 2 and 8 show results for continuous numeric variables, while other tables present results for categorical variables or were categorized for analysis. In addition to ICC, the 95% confidence interval (CI), the design effect (Deff) and the mean cluster size (n_a_), as well as the estimated prevalence (or mean) are presented. ICC ranged from <0.001 to 0.965, with a median of 0.028. ICC was < 0.1 in 78.5% of the variables and < 0.3 for 95% of them.

**Table 1:** **Estimates of prevalence (P), intracluster correlation coefficients (ICC), their respective 95% CI, design effect (Deff), and mean cluster size (n**_**a**_**) for categorical maternal characteristics**

**Variables**	**P (%)**	**ICC**	**95% CI for ICC**	**Deff**	**n**_**a**_
Skin color (white)	43.1	0.145	0.058-0.233	42.2	265
Marital status (with a partner)	77.7	0.008	<0.001-0.016	3.2	265
Schooling (>8 years)	60.5	0.030	0.008-0.053	10.6	261
Children under 5 years (≥1)	27.1	0.005	<0.001-0.011	2.4	265
Time since last delivery (until 12 months)	8.4	0.011	<0.001-0.022	2.8	155
Previous cerclage	1.1	0.001	<0.001-0.004	1.2	264
Previous preterm birth	17.3	0.007	<0.001-0.013	3.2	264
Previous preterm birth of multiples	1.0	<0.001	<0.001-0.003	1.2	264
Previous preterm labor	7.4	0.011	0.001-0.021	4.3	264
Previous prelabor PROM	7.2	0.002	<0.001-0.006	1.8	264
Previous indicated preterm birth	7.7	0.004	<0.001-0.009	2.0	263
Previous newborn weight < 2500 g	14.8	0.010	<0.001-0.019	4.1	262
**Previous chronic diseases:**					
● Chronic hypertension	8.2	0.004	<0.001-0.009	2.4	265
● Diabetes mellitus	2.1	0.010	0.001-0.019	3.6	265
● Thyroid disease	1.8	0.012	0.002-0.023	4.4	265
● Cardiac disease	1.3	0.002	<0.001-0.005	1.4	265
● Lung disease	2.9	0.006	<0.001-0.012	2.8	265
● Renal disease	1.8	0.013	0.002-0.024	4.7	265
● Digestive disease	1.3	0.009	0.001-0.018	3.3	265
● Hematological disease	1.4	0.012	0.002-0.023	4.7	265
● Neurological disease	1.2	0.008	<0.001-0.016	3.7	265
● Psychiatric disease	1.4	0.022	0.005-0.038	7.0	265
● HIV	1.3	0.006	<0.001-0.012	2.6	265
● Other	6.5	0.033	0.009-0.057	11.8	265

**Table 2:** **Estimates of mean, intracluster correlation coefficients (ICC), their respective 95% CI, design effect (Deff), and mean cluster size (n**_**a**_**) for numeric maternal characteristics**

**Variable**	**Mean**	**ICC**	**95% CI for ICC**	**Deff**	**n**_**a**_
Age (years)	26.1	0.018	0.004-0.033	5.3	265
Month stopped working	6.9	0.015	<0.001-0.032	2.6	99
Workload (hours daily)	8.0	0.040	0.007-0.072	6.9	98
Pre-pregnancy weight (Kg)	62.1	0.021	0.005-0.038	6.6	250
Height (m)	1.6	0.041	0.011-0.071	9.8	238
Final weight (Kg)	73.2	0.022	0.005-0.040	6.4	237
Weight gain in pregnancy (Kg)	10.9	0.012	0.001-0.023	4.5	229
Initial Body Mass Index (Kg/m^2^)	24.4	0.012	0.001-0.024	4.5	230
Final Body Mass Index (Kg/m^2^)	28.7	0.016	0.002-0.030	5.5	220
Number of pregnancies	2.4	0.006	<0.001-0.013	2.8	265
Number of vaginal deliveries	0.8	0.005	<0.001-0.011	2.5	265
Number of cesarean sections	0.3	0.014	0.002-0.025	4.7	265
Number of abortions	0.3	0.006	<0.001-0.013	2.3	265
Number of uterine curettage	0.2	0.008	<0.001-0.015	2.9	264

**Table 3:** **Estimates of prevalence (P), intracluster correlation coefficients (ICC), their respective 95% CI, design effect (Deff), and mean cluster size (n**_**a**_**) for maternal socio-demographic characteristics**

**Variable**	**P (%)**	**ICC**	**95% CI for ICC**	**Deff**	**n**_**a**_
Household (rural)	9.8	0.097	0.034-0.159	32.9	264
Homeownership	57.5	0.041	0.012-0.070	15.2	265
Paved street	78.7	0.181	0.077-0.286	60.0	262
Piped water	94.2	0.090	0.031-0.149	30.0	263
Sewer	86.8	0.191	0.083-0.300	53.8	261
Family income (>US$ 400.00)	38.8	0.103	0.037-0.168	28.8	244
Paid work	42.6	0.036	0.010-0.063	10.8	263
Paid work in pregnancy	88.8	0.041	0.008-0.073	7.4	112
Strenuous work	43.4	0.037	0.006-0.068	5.5	99
Standing work	61.4	0.017	<0.001-0.034	2.8	99
Night work	19.5	0.033	0.004-0.061	4.3	98
Housework (alone)	50.7	0.019	0.004-0.034	7.3	265

**Table 4:** **Estimates of prevalence (P), intracluster correlation coefficients (ICC), their respective 95% CI, design effect (Deff), and mean cluster size (n**_**a**_**) for categorical variables of process during pregnancy**

**Variable**	**P (%)**	**ICC**	**95% CI for ICC**	**Deff**	**n**_**a**_
Healthcare facility used for prenatal care:					
● Primary health care unit	71.3	0.117	0.044-0.191	31.1	256
● Hospital	34.3	0.185	0.079-0.291	46.2	256
● Private clinic	9.3	0.051	0.015-0.086	15.9	256
● Other	0.3	0.005	<0.001-0.011	2.4	256
● Without prenatal care	3.2	0.003	<0.001-0.008	2.2	256
Prenatal care by physician	89.7	0.195	0.085-0.305	64.5	256
Start of prenatal care (1^st^ trimester)	64.8	0.034	0.009-0.059	8.9	219
Number of prenatal care visits (≥6)	58.8	0.054	0.016-0.092	13.7	231
Ultrasound during prenatal care	98.4	0.001	<0.001-0.004	1.4	254
Physical effort	42.0	0.053	0.016-0.089	14.8	263
Depression	32.5	0.073	0.024-0.122	26.2	263
Anxiety	65.5	0.099	0.035-0.163	38.0	263
Smoking	13.5	0.020	0.004-0.036	7.7	265
Use of alcohol	15.9	0.031	0.008-0.054	10.2	263
Illicit drugs use (during or before)	4.9	0.015	0.002-0.027	5.8	265
Vaginal discharge treatment (self-reported)	36.6	0.010	0.001-0.020	4.1	264
Vulvovaginitis:					
● Bacterial vaginosis	12.9	0.039	0.008-0.069	11.1	160
● Candidiasis	13.5	0.061	0.016-0.106	11.2	160
● Trychomoniasis	1.4	0.011	<0.001-0.023	4.9	160
● Other vulvovaginitis	0.9	0.030	0.005-0.054	6.2	160
Vulvovaginitis treatment (registered)	24.1	0.073	0.020-0.126	15.1	164
Urinary infection treatment (self-reported)	36.3	0.018	0.004-0.032	6.7	261
Urinary infection (registered)	32.9	0.032	0.008-0.057	10.0	209
● Asymptomatic bacteriuria	15.7	0.084	0.027-0.140	23.2	184
● Cystitis	7.1	0.028	0.006-0.050	7.9	184
● Pyelonephritis	2.0	0.003	<0.001-0.008	2.0	184
Urinary treatment (registered)	2.1	0.075	0.023-0.126	18.2	184
Periodontal infection	17.0	0.036	0.010-0.063	14.5	262
Other infection	9.1	0.019	0.004-0.035	7.5	263
● Unknown fever	1.8	0.024	0.006-0.043	11.1	265
● Diarrhea fever	0.9	0.006	<0.001-0.012	3.2	265
● HIV - diagnosis in pregnancy	0.6	0.002	<0.001-0.006	2.1	265
● Pneumonia	0.5	<0.001	<0.001-0.003	1.2	265
● Tuberculosis	<0.1	<0.001	<0.001-0.003	0.8	265
● Sinusitis/tonsillitis	3.4	0.015	0.003-0.028	6.7	265
● Hepatitis	0.2	0.007	<0.001-0.014	4.2	265
● Genital herpes	<0.1	0.001	<0.001-0.004	1.4	265
● Toxoplasmosis	0.5	0.009	<0 001-0.018	3.4	265
Anemia	29.2	0.046	0.013-0.078	13.4	259
Iron replacement	84.9	0.037	0.001-0.063	12.1	264
Bleeding	23.9	0.012	0.001-0.022	4.6	264
● Bleeding in first trimester	12.2	0.006	<0.001-0.013	2.6	264
● Bleeding in second trimester	6.7	0.002	<0.001-0.006	1.6	264
● Bleeding in third trimester	6.3	0.013	0.002-0.024	6.1	264

**Table 5:** **Estimates of prevalence (P), intracluster correlation coefficients (ICC), their respective 95% CI, design effect (Deff), and mean cluster size (n**_**a**_**) for categorical variables of process during pregnancy**

**Variable**	**P(%)**	**ICC**	**95% CI for ICC**	**Deff**	**n**_**a**_
Hospitalization	22.3	0.030	0.008-0.052	10.0	265
Reasons for hospitalization:					
● Emesis	0.6	0.006	0.001-0.013	2.3	264
● Uterine contraction	5.7	0.014	0.002-0.026	5.3	264
● Amniorrhexis	2.2	0.009	0.001-0.017	4.0	264
● Bleeding	2.6	0.008	<0.001-0.016	3.0	264
● Maternal disease	8.9	0.029	0.007-0.050	10.1	264
● Fetal disease	0.8	0.028	0.007-0.049	6.0	264
Syphilis	1.6	0.004	<0.001-0.009	1.7	265
Anemia (registered)	32.0	0.070	0.023-0.118	24.1	238
Treatment for anemia	52.6	0.283	0.138-0.428	74.8	213
Short cervix (US)	1.4	0.011	<0.001-0.022	4.0	209
Cervical insufficiency	2.1	0.005	<0.001-0.012	2.6	230
Cerclage	1.4	0.019	0.003-0.034	5.6	238
Uterine anomalies	0.6	<0.001	<0.001-0.003	0.6	237
Fibroid	1.9	0.002	<0.001-0.006	1.5	233
Maternal diseases:					
● Diabetes	5.7	0.027	0.006-0.047	7.8	254
● Gestational hypertension	7.7	0.025	0.006-0.045	9.4	254
● Preeclampsia/eclampsia/HELLP	16.2	0.062	0.019-0.104	22.5	254
● Chronic hypertension	5.7	0.007	<0.001-0.014	2.8	254
● Other chronic infection	0.7	0.010	0.001-0.020	4.5	254
● Thyroid diseases	1.6	0.027	0.006-0.047	8.2	254
● Renal disease	1.2	0.008	<0.001-0.015	3.1	254
● Sickle cell anemia	0.3	0.002	<0.001-0.006	1.5	254
● Other chronic anemia	0.5	<0.001	<0.001-0.003	0.7	254
● Cardiac disease	1.1	0.003	<0.001-0.008	1.9	254
● Lung disease	1.5	0.009	<0.001-0.017	3.8	254
● Epilepsy	0.6	0.001	<0.001-0.004	1.5	254
● Systemic lupus erythematous	0.5	0.020	0.004-0.036	4.6	254
● Other collagenoses	0.2	0.001	<0.001-0.004	1.4	254
● Digestive disease	0.6	0.006	<0.001-0.013	3.1	254
● Bariatric surgery	<0.1	<0.001	<0.001-0.003	0.8	254
● Psychiatric disease	1.0	0.015	0.003-0.028	5.4	254
● Orthopedic disease	0.2	<0.001	<0.001-0.003	0.9	254
● Neoplasms	0.2	0.001	<0.001-0.004	1.4	254
● Thrombosis or thrombophilia	0.4	0.006	<0.001-0.013	2.4	254
Fetal malformation	5.5	0.146	0.057-0.236	35.9	246
Fetal growth restriction	9.3	0.019	0.004-0.035	6.9	246
Other fetal morbidity	7.4	0.386	0.219-0.554	101.5	246
Triplets	2.0	<0.001	<0.001-0.030	1.0	22
Infertility treatment	4.4	<0.001	<0.001-0.031	0.9	22
Multiple monochorionic pregnancy	35.8	0.046	<0.001-0.111	2.0	18
Multiple monoamniotic pregnancy	5.8	0.038	<0.001-0.098	1.9	18
Twin-to-twin transfusion syndrome	5.4	<0.001	<0.001-0.036	0.9	18

**Table 6:** **Estimates of prevalence (P), intracluster correlation coefficients (ICC), their respective 95% CI, design effect (Deff), and mean cluster size (n**_**a**_**) for categorical variables of process during labor**

**Variable**	**P(%)**	**ICC**	**95% CI for ICC**	**Deff**	**n**_**a**_
Mode of onset of labor (spontaneous)	55.3	0.018	0.004-0.032	6.5	265
Intrapartum antibiotic (ATB)	51.8	0.194	0.084-0.304	71.8	260
● ATB for fever	0.5	0.003	<0.001-0.008	1.8	252
● ATB for GBS colonization	1.9	0.019	0.004-0.034	5.6	252
● ATB for risk factor to GBS	20.0	0.148	0.058-0.238	48.2	252
● ATB for other reasons	29.1	0.384	0.217-0.550	148.0	252
Analgesics during labor:					
● Epidural	4.2	0.200	0.087-0.313	43.3	259
● Epidural plus spinal anesthesia	3.7	0.201	0.088-0.314	74.3	259
● Spinal anesthesia	20.1	0.338	0.181-0.495	112.8	259
● Meperidine	0.8	0.018	0.004-0.033	6.6	259
● Tramadol	0.2	0.002	<0.001-0.006	1.4	259
● Benzodiazepines	0.1	0.008	<0.001-0.017	3.6	259
● Antispasmodics	2.2	0.071	0.023-0.119	21.2	259
● Oral analgesics	2.0	0.091	0.031-0.150	23.0	259
● Other analgesics	2.4	0.102	0.036-0.168	46.6	259
Mode of delivery (vaginal)	48.8	0.024	0.006-0.043	7.7	265
Episiotomy	38.7	0.176	0.068-0.283	31.5	126
Forceps	3.9	0.056	0.014-0.099	12.9	116
Cesarean indication:					
● Fetal distress	25.7	0.016	0.001-0.031	3.8	133
● Cephalic-pelvic disproportion	2.8	0.016	0.001-0.032	3.2	133
● Two or more cesarean scars	9.8	0.006	<0.001-0.014	2.0	133
● Pelvic or other abnormal fetal presentation	15.6	0.012	<0.001-0.025	2.9	133
● Functional dystocia	2.2	0.022	0.003-0.041	3.8	133
● Diabetes	1.8	0.013	<0.001-0.027	3.3	133
● Arterial hypertension	22.7	0.043	0.011-0.075	7.4	133
● Cardiac disease	0.6	0.009	<0.001-0.020	1.6	133
● HIV	1.6	0.005	<0.001-0.012	1.7	133
● Placenta previa	2.0	0.006	<0.001-0.014	1.6	133
● Abruptio placentae	4.8	0.005	<0.001-0.013	1.9	133
● Uterine rupture	0.1	0.006	<0.001-0.015	1.1	133
● Fetal malformation	3.2	0.133	0.051-0.215	18.9	133
● Fetal macrosomia	1.7	0.002	<0.001-0.008	1.4	133
● Maternal choice	1.0	0.037	0.008-0.065	7.2	133
● Other	17.1	0.082	0.027-0.137	14.9	133
Type of incision (segmental transverse)	96.3	0.193	0.081-0.304	13.5	126

**Table 7:** **Estimates of prevalence (P), intracluster correlation coefficients (ICC), their respective 95% CI, design effect (Deff), and mean cluster size (n**_**a**_**) for categorical newborn outcome variables**

**Variable**	**P(%)**	**ICC**	**95% CI for ICC**	**Deff**	**n**_**a**_
Diagnosis of gestational age (US)	45.4	0.264	0.128-0.399	84.8	265
Stillborn	3.1	0.026	0.006-0.046	7.5	265
Intubation at delivery	13.4	0.013	0.002-0.024	4.1	248
Use of surfactant	12.6	0.015	0.002-0.027	4.4	245
Fetal malformation	9.5	0.078	0.026-0.130	19.6	246
Ventilatory support	42.6	0.041	0.011-0.070	15.1	249
Neonatal morbidity	60.3	0.126	0.047-0.205	33.4	248
● Sepsis	27.7	0.051	0.011-0.091	8.3	144
● Respiratory distress	73.4	0.061	0.014-0.107	9.9	148
● Pneumothorax	3.6	0.041	0.007-0.075	8.2	141
● Cerebral hemorrhage (1–4)	8.7	0.052	0.007-0.097	5.8	114
● Lung hemorrhage	3.7	0.028	0.004-0.053	5.7	143
● Hematologic dysfunction	51.0	0.267	0.116-0.417	71.7	146
● Endocrine dysfunction	22.0	0.119	0.036-0.201	30.3	145
● Renal dysfunction	6.4	0.013	<0.001-0.027	3.5	145
● Immune dysfunction	6.5	0.092	0.025-0.158	22.1	145
● Musculoskeletal morbidity	8.6	0.190	0.071-0.310	38.4	146
● Gastrointestinal dysfunction	43.2	0.340	0.168-0.512	70.6	146
● Hypovolemia	10.4	0.026	0.003-0.049	6.0	146
● Necrotizing enterocolitis	2.4	0.020	0.001-0.038	3.2	145
● Convulsion/anticonvulsants	4.8	0.039	0.007-0.071	6.7	146
● Vasoactive amines	12.2	0.019	0.001-0.037	3.5	146
● Pneumonia	5.6	0.118	0.036-0.200	15.6	145
● Oxygen therapy with 28 days	8.0	0.021	0.002-0.041	3.8	145
● Oxygen therapy with 56 days	2.9	0.012	<0.001-0.025	2.8	143
● Degree of retinopathy (1–3)	4.8	0.028	<0.001-0.056	4.2	99
Condition at discharge (live)	91.8	0.014	0.002-0.026	4.1	252

**Table 8:** **Estimates of mean, intracluster correlation coefficients (ICC), their respective 95% CI, design effect (Deff), and mean cluster size (n**_**a**_**) for numeric newborn outcome variables**

**Variable**	**Mean**	**ICC**	**95% CI for ICC**	**Deff**	**n**_**a**_
Gestational age (weeks)	34.5	0.031	0.008-0.055	10.4	265
Birth weight (g)	2321.1	0.033	0.009-0.058	11.6	264
Birth weight 2° twin (g)	1905.2	0.007	<0.001-0.043	1.4	21
APGAR 1^st^ minute	7.3	0.032	0.008-0.056	8.6	261
APGAR 1^st^ minute 2° twin	6.7	0.042	<0.001-0.098	2.2	21
APGAR 5^th^ minute	8.6	0.041	0.012-0.070	11.5	261
APGAR 5^th^ minute 2° twin	8.3	0.002	<0.001-0.034	1.1	21
Head circumference (cm)	31.7	0.031	0.008-0.055	10.1	236
Head circumference 2° twin (cm)	30.8	0.018	<0.001-0.067	1.1	18
Stature (cm)	44.3	0.031	0.007-0.054	10.3	237
Stature 2° twin (cm)	42.3	0.025	<0.001-0.077	1.4	18
Length of ICU stay (days)	8.4	0.088	0.028-0.148	21.4	220
Length of hospital stay (days)	13.3	0.037	0.009-0.065	8.5	235
Age of newborn at sepsis (days)	4.6	0.173	0.054-0.292	7.2	39
Age of newborn at death (days)	8.9	0.088	<0.001-0.179	2.7	17

**Table 9:** **Estimates of prevalence (P), intracluster correlation coefficients (ICC), their respective 95% CI, design effect (Deff), and mean cluster size (n**_**a**_**) for categorical management variables in spontaneous labor conditions or preterm due to pPROM**

**Variable**	**P(%)**	**ICC**	**95% CI for ICC**	**Deff**	**n**_**a**_
**Preterm birth due to spontaneous labor:**					
Use of corticosteroids	28.5	0.032	0.002-0.062	5.2	73
Corticosteroids (betamethasone)	86.4	0.851	0.754-0.948	18.8	21
Use of tocolytic agents	23.6	0.068	0.015-0.121	8.7	72
Association of tocolytic agents	9.9	0.368	0.167-0.570	8.4	17
Therapeutic failure of tocolysis	11.4	0.165	0.029-0.301	4.3	17
Use of magnesium sulphate (neuroprotection)	3.9	0.070	0.016-0.125	9.3	70
Use of antibiotics	42.8	0.262	0.117-0.407	28.8	72
Intravenous antibiotic	93.3	0.321	0.127-0.515	10.1	31
Association of antibiotic	15.3	0.144	0.025-0.263	12.9	30
Group B streptococcus screening	24.3	0.286	0.131-0.442	26.2	65
**Preterm birth due to pPROM:**					
Use of corticosteroids	40.5	0.042	0.002-0.083	3.7	53
Corticosteroids (betamethasone)	85.0	0.965	0.941-0.990	23.5	21
Use of tocolytic agents	17.7	0.547	0.364-0.729	38.3	56
Use of antibiotics	78.2	0.233	0.095-0.371	18.3	54
Intravenous antibiotic	91.0	0.366	0.180-0.552	14.4	41
Association of antibiotic	20.9	0.245	0.093-0.397	20.1	41
Group B streptococcus screening	36.3	0.441	0.260-0.622	27.9	50
Hydration solution (saline)	11.0	0.419	0.235-0.602	20.1	52

**Table 10:** **Estimates of prevalence (P), intracluster correlation coefficients (ICC), their respective 95% CI, design effect (Deff), and mean cluster size (n**_**a**_**) for diagnosis and management among categorical variables related to therapeutic preterm delivery**

**Variable**	**P(%)**	**ICC**	**95% CI for ICC**	**Deff**	**n**_**a**_
Therapeutic delivery for maternal disease	74.6	0.102	0.032-0.172	9.6	73
Therapeutic delivery for fetal disease	54.1	0.065	0.016-0.115	7.0	73
Maternal disease responsible for interruption of pregnancy:					
● Diabetes	7.3	0.063	0.011-0.115	5.5	54
● Gestational hypertension	12.9	0.144	0.048-0.240	9.4	54
● Chronic hypertension	15.3	0.009	<0.001-0.027	1.8	54
● Preeclampsia	58.2	0.079	0.017-0.140	5.4	54
● Eclampsia	3.2	0.017	<0.001-0.041	1.8	54
● HELLP syndrome	9.4	0.012	<0.001-0.031	1.2	54
● Abruptio placentae	7.7	0.009	<0.001-0.026	1.6	54
● Previous placentae	3.3	0.001	<0.001-0.013	0.8	54
Fetal disease responsible for interruption of pregnancy:					
● Fetal distress	32.6	0.052	0.010-0.095	6.1	71
● Fetal growth restriction	19.8	0.037	0.004-0.069	4.4	71
● Malformation	5.2	0.144	0.052-0.236	13.5	71
● Other fetal condition	15.1	0.161	0.061-0.262	15.8	71
Exams to evaluate fetal condition:					
● Cardiotocography	61.0	0.299	0.148-0.451	23.7	67
● Dopplerfluxometry	61.1	0.159	0.059-0.260	14.4	67
● Fetal biophysical profile	32.2	0.508	0.331-0.686	43.5	67
● Fetal movements control	4.3	0.101	0.030-0.172	12.3	67
● Other exam	12.9	0.041	0.005-0.077	4.1	67
Determinant exams for diagnosis:					
● Cardiotocography	23.2	0.104	0.032-0.176	10.1	70
● Dopplerfluxometry	29.8	0.064	0.014-0.113	6.4	70
● Fetal biophysical profile	14.6	0.290	0.142-0.438	24.0	70
● Fetal echocardiography	1.2	0.030	0.001-0.058	4.6	70
● Maternal hepatic dysfunction	15.9	0.194	0.079-0.308	19.9	70
● Maternal hematologic dysfunction	21.0	0.278	0.134-0.423	29.9	70
● Other	41.2	0.102	0.031-0.173	10.0	70
Maternal or fetal attempted treatment	57.9	0.056	0.012-0.101	5.7	71
Use of corticosteroids	42.6	0.059	0.012-0.105	6.2	70
Maternal condition at hospital discharge (cured)	24.8	0.153	0.057-0.249	15.7	73

Tables 1 and 2 presents some variables related to maternal characteristics, including clinical and obstetrical history. ICCs ranged from <0.001 to 0.145 (median 0.011). Table 3 shows the socio-demographic variables studied, and ICC ranged from 0.017 to 0.191 (median 0.041). Tables 4 and 5 presents variables related to pregnancy characteristics with ICC ranging from 0.001 to 0.386 (median 0.015). The variables related to labor conditions were presented in Table 6. It can be observed that ICC ranged from 0.002 to 0.384, with a median of 0.022. Tables 7 and 8 shows variables related to perinatal outcomes and ICC were < 0.1 in 81% of them. The most important outcome variable, newborn morbidities, is presented in Table 7. Tables 9 and 10 present some variables analyzed specifically for preterm births and are related to management. Most variables in Table 9 showed ICC greater than 0.3 and the greatest ICC of this study (0.965) was relative to the variable “corticosteroids use”, a management aspect well defined and well-established in all obstetric protocols, so there were high degree of homogeneity in clusters in these variables. The median of ICCs was 0.274. The median ICC in Table 10 was 0.079.

### Estimated deffs

Estimated Deffs are presented in Tables 1, 2, 3, 4, 5, 6, 7, 8, 9 and 10 for each of 261 variables. Deff ranged from 0.6 to 148.0, with a median of 6.1.

Deff were under 5.0 in 74% of variables in Tables 1 and 2, ranging from 1.2 to 42.2 (median 3.65). Table 3 presents Deff values ranging from 2.8 to 60 (median 13.0). In variables related to gestational process (Tables 4 and 5), Deff values ranged from 0.6 to 101.5 (median 4.9). The variables related to labor conditions (Table 6) showed Deff ranging from 1.1 to 148 (median 6.6), with 60% of them under 8.0. In Tables 7 and 8, related to perinatal outcomes, Deff values ranged from 1.1 to 84.8 (median 7.8). Tables 9 and 10 presented Deff median of 16.35 and 7.0, respectively.

We can observe that greater Deff median is present in process variables (Table 9), and greater ICCs.

## Discussion

This study presents a large number of intracluster correlation coefficients whose values can be considered low (close to zero) in most variables, showing intracluster heterogeneity.

The greater ICC values were found in process variables, especially management in spontaneous preterm labor conditions, as corticosteroids use, Group B streptococcus screening, use of tocolytic agents and use of antibiotic. Indeed, the mean ICC value for these variables was 10 times higher than the mean ICC of the study. The variable with the highest ICC was “corticosteroids – betamethasone”, with a value of 0.965. The prevalence of this variable was 85%, showing a high degree of homogeneity in this management for preterm labor. These findings are in accordance with the literature that describes ICC values generally higher for variables related to process compared to those variables related to outcome [15,16].

In the field of maternal and perinatal healthcare, Taljaard *et al.* calculated ICC values based on data obtained from secondary/tertiary services [16]. Comparing with our study, they found an overall median ICC of 0.067 versus 0.028. For maternal and newborn outcome variables, their median ICCs was 0.011 (versus 0.014), and 0.054 (versus 0.041), respectively. The findings of those investigators showed that, for variables associated to process, ICC values tend to be > 0.07. The present findings are in agreement with this observation.

Pagel *et al.*[17] estimated ICC for a range of outcomes using data from five community-based clusters randomized controlled trials in three low-income countries. Estimated ICC values for mortality outcomes were lower than those for process outcomes, with narrower confidence intervals throughout for trials with larger number of clusters.

All comparisons show that the smaller the cluster size, the higher the ICC and the opposite occurs regarding the prevalence of the condition. Estimates of intracluster correlation are much less reliable for rare outcomes and the size of the cluster had a greater impact than the number of clusters on the reliability of estimates for rare outcomes [17].

Furthermore, higher healthcare levels tend to increase the degree of homogeneity [18,19]. The size of ICC increases if the ICC represents data from secondary rather than primary care. This may be a reflection of the underlying heterogeneity of the datasets under consideration as the conditions represented across the different datasets were diverse. Although numerically small (average 0.01), such differences can have a substantial effect on sample size, even when the average of cluster is small [15]. The clusters in this study are secondary and tertiary hospitals, most of them are teaching hospitals, with the majority of procedures performed in conformity with evidence-based healthcare protocols.

Stratified randomization had the effect of reducing estimates of cluster correlation [15]. However, in the same way that in Brazilian Network for Surveillance of Severe Maternal Morbidity Study [20], which found ICC values close to zero, the selection of clusters did not performed stratification by region. The distribution of centers in this study, with almost half located in southeast region, is in accordance with the actual distribution of healthcare institutions and the proportionality of births per region in the country [21,22].

The large number of intracluster correlation coefficients presented in this study, considered low (close to zero) in most of variables, can probably be seen as a good parameter of variance for calculating sample size in new studies in the field of perinatal and maternal health [15].

We can, however, to identify some possible limitations of the study, including the fact that we used a non-probabilistic sample from the centers (hospitals). Therefore, strictly speaking, the findings cannot be generalized to other populations. However, the majority of hospitals included in the study was third level referral hospitals taking care of high risk pregnancies and preterm babies. Probably the results would be applicable to other centers with such characteristics, irrespective of being private or public, especially in middle income countries like Brazil.

## Conclusions

The Brazilian Multicenter Study on Preterm Birth, developed as part of the Brazilian Network for Studies on Reproductive and Perinatal Health, to the best of our knowledge is the first cross sectional multicenter study on this topic in the country. It represents a planned comprehensive assessment of preterm birth in Brazil and ICC values calculation and analysis of more than 250 maternal and newborn variables, showed heterogeneity of data in selected clusters. These findings increase reliability of study estimates and allow the use of these results to calculate the required sample size for future research studies in maternal and perinatal health.

## Abbreviations

CRF: Clinical research form; Deff: Design effect; ICC: Intraclass correlation coefficient; SRS: Simple random sampling.

## Competing interests

The authors declare that they have no competing interests.

## Authors’ contributions

The idea for the study first arose in a discussion among RPJ, JGC and RPT and then was developed and implemented by the whole research team. The first version of the manuscript was drafted by GJL and SMH, and then complemented with the suggestions of the others. RPJ and JGC supervised the entire process. All authors contributed to the development of the study protocol and approved the final version of manuscript.

## Pre-publication history

The pre-publication history for this paper can be accessed here:

http://www.biomedcentral.com/1471-2288/14/54/prepub
